# The Technology Acceptance of Video Consultations for Type 2 Diabetes Care in General Practice: Cross-sectional Survey of Danish General Practitioners

**DOI:** 10.2196/37223

**Published:** 2022-08-30

**Authors:** Daniel Cæsar Torp, Annelli Sandbæk, Thim Prætorius

**Affiliations:** 1 Steno Diabetes Center Aarhus Aarhus University Hospital Aarhus Denmark

**Keywords:** video consultations, telemedicine, diabetes, chronic diseases, general practice, technology acceptance, technology acceptance model

## Abstract

**Background:**

During the COVID-19 pandemic, video consultations became a common method of delivering care in general practice. To date, research has mostly studied acute or subacute care, thereby leaving a knowledge gap regarding the potential of using video consultations to manage chronic diseases.

**Objective:**

This study aimed to examine general practitioners’ technology acceptance of video consultations for the purpose of managing type 2 diabetes in general practice.

**Methods:**

A web-based survey based on the technology acceptance model measuring 4 dimensions—perceived usefulness, perceived ease of use, attitude, and behavioral intention to use—was sent to all general practices (N=1678) in Denmark to elicit user perspectives. The data were analyzed using structural equation modeling.

**Results:**

The survey sample comprised 425 general practitioners who were representative of the population. Structural equation modeling showed that 4 of the 5 hypotheses in the final research model were statistically significant (*P*<.001). Perceived ease of use had a positive influence on perceived usefulness and attitude. Attitude was positively influenced by perceived usefulness. Attitude had a positive influence on behavioral intention to use, although perceived usefulness did not. Goodness-of-fit indices showed acceptable fits for the structural equation modeling estimation.

**Conclusions:**

Perceived usefulness was the primary driver of general practitioners’ positive attitude toward video consultations for type 2 diabetes care. The study suggests that to improve attitude and technology use, decision-makers should focus on improving usefulness, that is, how it can improve treatment and make it more effective and easier.

## Introduction

### Background

Technological change and the use of new technologies in health care are driven by objectives to increase access to health care, reduce care costs, coordinate health care, and facilitate chronic disease prevention and management [1]. The COVID-19 pandemic, caused by SARS-CoV-2 infection, has spurred health care systems to rapidly change from delivering in-person care to using different types of web-based care [2-4] such as video consultations [5]. Within the primary care sector, the uptake of video consultations has increased [6], and general practitioners’ use of the technology has internationally moved from being used in pilot projects to wider-scale use [7-9]. The care potential of using video consultations in general practice is considered high [10,11], and this technology holds the potential to disrupt how health care is delivered in the primary care sector [12].

The recent uptake of video consultations in general practice is intriguing as the use of new health care technology and its implementation typically takes years [5,13]. This is because digital-first approaches to primary care could increase general practice workload [14] or threaten professional autonomy [15]. Similar to the hospital sector [16,17], knowledge about the impact of video consultations on general practice is in its infancy, and the literature is particularly short on quantitative studies [18]. The nascent literature finds that offering video consultations constitutes a significant change in how health care professionals deliver and patients receive care [19]. Research into factors that influence the implementation of video consultations in routine practice finds that, for instance, training is an important facilitator [20], and hesitance to change is an equally important barrier [21]. Research suggests that general practitioner characteristics (eg, age and sex) do not influence use, although working in larger practices makes it more likely [22,23]. Interaction and communication between patients and general practitioners during video consultations are usually effective [24,25]. However, patients and practitioners report mixed user experiences but with the important point that user ratings depend on the context in which video consultations are used [26-31]. Younger patients were found to be more likely to request or be offered a web-based visit [32].

However, research has not systematically elicited general practitioners’ attitudes toward video consultations or their perceptions of the ease of use or usefulness in general practice. This research gap is unfortunate as it is well established in IT literature that attitude and perception influence physicians’ use of other types of health care technology such as electronic patient records or telemedicine [33-35]. The technology acceptance model (TAM) has proven to be a robust model through rigorous empirical testing within and beyond health care [36,37]. TAM is capable of studying user attitudes and perceptions and has good predictive power of health technology use [38]. Central to the original TAM [39] and later extensions [40] is that the behavioral intention (BI) to use technology is influenced by users’ ratings of perceived usefulness (PU), perceived ease of use (PEOU), and attitude toward the technology. Importantly, BI to use predicts actual user behavior [41,42].

Using the insight that chronic disease prevention and management are key drivers of technological change, this paper studies the potential of using video consultations in general practice to manage type 2 diabetes for 3 reasons. First, type 2 diabetes is a chronic disease for which video consultation appears promising in general practice [43-45]. Second, previous research on the use of video consultations in general practice has mostly studied acute or subacute or out-of-hours care and, to a much lesser extent, the management of chronic care taking place during regular hours [17,25,31]. Third, it is important to find care models capable of delivering high-quality and efficient type 2 diabetes care in general practice [46,47] as the disease prevalence is increasing [48] and people living with type 2 diabetes are at higher risk of developing complications [49].

The aim of this paper is to use TAM to study general practitioners’ technology acceptance of video consultations to manage type 2 diabetes in general practice. The hypotheses were that higher levels of attitude, PU, and PEOU positively affect general practitioners’ BI to use video consultations to manage type 2 diabetes. Bringing to bear TAM on video consultations in general practice allows exploring the potential of using the technology for a type of chronic care where health care systems need to find new ways of increasing health care access and cutting care costs.

### Research Model and Hypotheses

**Figure 1:**
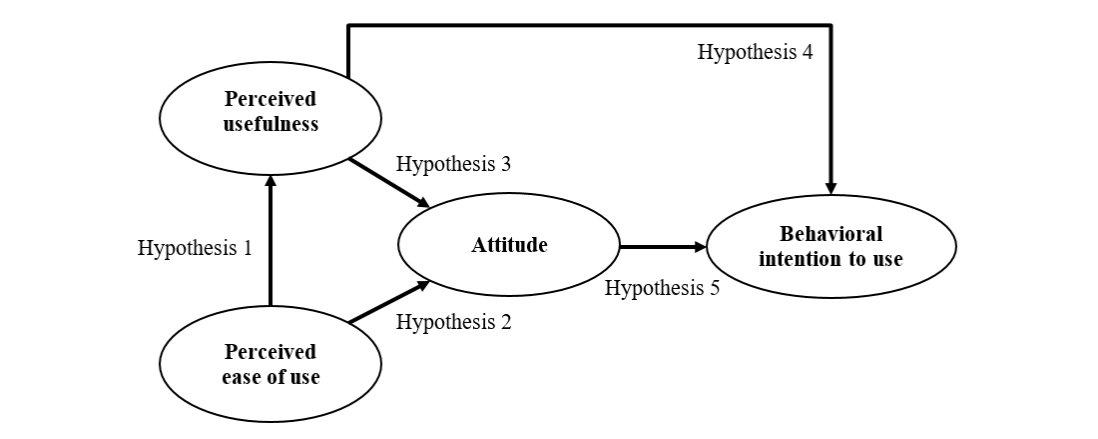
Research model based on the technology acceptance model.

The research model (Figure 1) builds on TAM [39] and posits that general practitioners’ perception of the degree to which video consultations used to manage type 2 diabetes are easy to use affects both perceptions of usefulness and attitudes toward using the technology. General practitioners’ attitudes are also influenced by their perception of how useful the technology is. Ultimately, general practitioners’ intention to use video consultations to manage type 2 diabetes can be explained by their attitude toward the technology and PU. The following develops 5 hypotheses by combining research insights on TAM, general practitioners, and the primary health care domain.

PEOU influences BI to use indirectly through both attitude and PU. A high PEOU represents the belief that using the technology will require little to no effort [39]. PU concerns the extent to which a user believes that the technology can improve or make their work more effective and easier and how it will be advantageous over the current practice. The relationship between PEOU and PU is expected to be positive as health care studies find that a higher level of PEOU leads to higher ratings of P [50-52]. Moreover, studies have shown that when a technology is perceived as easy to use, the attitude toward the technology is more positive [40,52]. The attitudinal component of the model measures an individual’s affective response to adopting a new technology. Attitude concerns the extent to which a user finds that using the technology is a good idea, beneficial, or unpleasant for the way they work [39]. PU is considered particularly important in general practice [53,54], and research using TAM finds that physicians’ PU influences attitudes toward health care technology [55,56]. Thus, 3 hypotheses about PEOU, PU, and attitude were formed:

- Hypothesis 1: PEOU has a positive impact on the PU of video consultations for type 2 diabetes care.
- Hypothesis 2: PEOU has a positive impact on attitudes toward video consultations for type 2 diabetes care.
- Hypothesis 3: PU has a positive impact on attitude toward video consultations for type 2 diabetes care.

The BI to use represents an individual’s intention to use a new technology [41]. BI to use is an important component as it is a proxy capable of predicting subsequent actual user behavior in health care and beyond [33,41,42]. According to TAM, the extent to which users perceive a technology to be useful is directly influenced by their ratings of BI to use [38]. In the context of general practice, research has found a positive relationship between PU and BI to use [35,57-59]. Similarly, TAM suggests that the attitude of a user manifests itself as a positive or negative view of the BI to use technology. Research in the domain of primary health care finds that attitude influences the BI to use health care technology [23,60,61]. Thus, 2 hypotheses about PU, attitude, and BI to use were formulated:

- Hypothesis 4: PU has a positive impact on the BI to use video consultations for type 2 diabetes care.
- Hypothesis 5: Attitude toward video consultations for type 2 diabetes care has a positive impact on the BI to use the technology.

## Methods

### Research Design and Setting

Data were collected through a cross-sectional web-based survey distributed to all general practitioners in Denmark (n=3326). The Danish health care system is mostly tax financed, and citizens can receive care from general practice free of per service charge. Danish general practitioners are self-employed but work on contracts for the public funder. Most general practitioners work in partnership practices, and their income is generated as a combination of fee for service and capitation [62]. The incentive for Danish general practitioners to use video consultations increased during the COVID-19 pandemic because of an agreement between the General Practitioners’ Organization (negotiating on behalf of Danish general practitioners) and the Danish Regions (responsible for procuring health services), which agreed on a fee for service to general practitioners to provide video consultations to patients.

### Survey Measures

The main measures (13 items) central to our hypotheses originated from TAM [39] and health care studies [55] to ensure the validity of the measures. The measures were adapted to the specific context of general practice and video consultations, translated into Danish, and repeatedly examined to ensure consistency. PU, attitude, and BI to use were measured using 3 items each, and PEOU was measured using 4 items (Textbox 1). An item each in the attitude and BI to use dimensions was negatively worded to reduce the risk of agreement bias [55]. All items were measured on 5-point Likert scales, with scores ranging from 1 (strongly disagree) to 5 (strongly agree). For PEOU, the items were worded according to the user status of the respondent (user vs nonuser of video consultations) to make the formulation relevant to the respondent. Respondents were able to skip questions or choose *do not know* (the latter being treated as missing data in subsequent analyses). Demographic measures (12 items) such as age and sex were collected to analyze the representativeness of the study sample in comparison with the total population of general practitioners. Before distribution and to test face validity, the survey was evaluated and revised according to inputs from 5 general practitioners working in each of the 5 Danish Regions.

**Items used in the research model.**

- Perceived usefulness (PU)
  PU1: can *improve* my treatment
  PU2: can make my treatment more *effective*
  PU3: can make my treatment *easier*
- Perceived ease of use (PEOU; worded differently for nonusers of video consultations as illustrated in brackets)
  PEOU1: *learning* to use was (would be) easy
  PEOU2: *(would be) easy to get* software to do what I need
  PEOU3: (would be) easy to *master*
  PEOU4: (would be) easy to *use*
- Attitude (ATT)
  ATT1: using is a *good idea*
  ATT2: using is *unpleasant*
  ATT3: using is *beneficial*
- Behavioral intention (BI)
  BI1: intend to use as *often as possible*
  BI2: even when possible, *do not intend* to use
  BI3: would *use to the extent possible*

### Recruitment and Data Collection

The survey was administered using SurveyXact (Rambøll Management) [63]. To identify general practices, a list of all 1718 general practices in Denmark was obtained from MedCom (a provider of Danish public health care systems) [64] in January 2021. Of these 1718 practices, 44 (2.56%) general practices were excluded as they were managed by parties outside the target group of our study (eg, by Danish Regions). In total, 1674 general practices, representing 3326 general practitioners, were available for distribution [65].

The survey was distributed to general practices as an electronic letter on January 7, 2021, via the Danish public electronic mailbox system (e-Boks Business) using publicly available data from MedCom. The letter contained information about the study and a survey link. Participants were informed about data protection measures, anonymity of participation, and the option to be paid—DKK 276.72 (US $44) based on a General Practitioners'’ Organization tariff—for the 20 minutes it maximally takes to complete the survey. The letter was addressed to the clinic, and all trained general practitioners were encouraged to participate. Unfortunately, it was not possible to contact each general practitioner directly as this information was not publicly available. The survey link was open and only available in a letter to ensure anonymity and availability for all general practitioners in a clinic. Data entry for payments was conducted in a separate survey to preserve anonymity. Two reminders were sent on January 21, 2021, and February 2, 2021. The data collection ended on February 7, 2021.

The Committee of Multipractice Studies in General Practice (journal number 25-2020) evaluated the study and recommended that general practitioners participate in the survey. This study was reported to the Danish Data Protection Agency (journal number 1-16-02-343-20).

### Ethics Approval

The Research Ethics Committees for Central Denmark Region (1-10-72-181-20) concluded that the study could be conducted without approval from the committee as “According to the Consolidation Act on Research Ethics Review of Health Research Projects, Consolidation Act number 1083 of 15 September 2017, section 14(2) notification of questionnaire surveys or medical database research projects to the research ethics committee system is only required if the project involves human biological material*.*”

### Data Analysis

Data were analyzed using Stata (version 17.0; StataCorp) [66]. To compare sample demographics with the population of general practitioners, we analyzed the latter using registry data made available by the Danish Health Data Authority [67]. The measures used in TAM were analyzed for normality distribution, internal consistency, convergent validity, and discriminant validity. Normality was examined by calculating skewness, kurtosis, and the Mardia multivariate kurtosis test. Internal consistency was assessed using Cronbach α with an acceptable threshold of .70 [68]. Confirmatory factor analysis was performed to determine model validity. Factor loadings of ≥0.7 were deemed acceptable [69]. Subsequently, we explored the research model using structured equation modeling [70], which is standard in the data analysis of TAM [37]. We used quasi-maximum likelihood as the estimator, with Satorra-Bentler adjustments because of our findings of nonnormality for some of the measures [71]. *P*<.05 was set as the threshold for statistical significance.

We report the unstandardized and standardized path coefficients from structured equation modeling. The unstandardized path coefficients reflect the expected (linear) change in the dependent variable with each unit change in the independent variable, given the other variables in the model. The standardized path coefficients express relationships in the same unit; that is, SDs. The interpretation is that when an independent variable (eg, PU) changes by 1 SD, then the dependent variable (eg, BI to use) changes by an SD as well. By placing all coefficients in the same unit, the SDs for different variables measured in different metrics become interpretationally equivalent.

## Results

### Demographic Characteristics

A total of 457 general practitioners answered the survey, from which 32 (7%) incomplete responses were excluded, resulting in 425 (93%) respondents. The sample represented 12.78% (425/3326) of all Danish general practitioners. The sample represented 18.82% (315/1674) of Danish general practices. Compared with the population of general practitioners, Pearson chi-square tests showed that the individual characteristics of the study sample (ie, sex and age groups) were representative of the population not participating (Table 1). The sample differed with regard to general practice characteristics (ie, clinic and municipality type) as general practitioners from more partnership practices participated than from solo practices, and a larger share of general practitioners working in practices in the capital area participated. The incomplete responses had similar demographics to the complete responses, with most (23/32, 72%) dropping out during or directly after the demographic items.

**Table 1:** Overview of respondents in sample and comparison with the remaining population.

Characteristics^a^		Survey sample (n=425), n (%)	Population not in the sample (n=2901), n (%)	Pearson chi-square (*df*)
Sex (female)^b^		226 (53.1)	1659 (57.1)	0.2 (1)
**Age group (years)^b^**				0.8 (6)
	30-39	26 (6.3)	205 (7.1)	
	40-44	75 (18.1)	577 (20)	
	45-49	100 (24.2)	614 (21.2)	
	50-54	59 (14.3)	416 (14.4)	
	55-59	64 (15.5)	433 (15)	
	60-64	57 (13.8)	387 (13.4)	
	≥65	33 (8)	260 (9)	
**Municipality type where general practitioners work^c,d^**				0.0 (4)
	Capital area	133 (31.3)	789 (25.5)	
	Large city	63 (14.8)	392 (12.7)	
	Province city	88 (20.7)	754 (24.4)	
	Suburban	70 (16.5)	507 (16.4)	
	County	71 (16.7)	654 (21.1)	
**Clinic type^c^**				<0.001 (2)
	Solo clinic	105 (25.1)	447 (35.7)	
	Cooperation clinic	52 (12.4)	145 (11.6)	
	Partnership clinic	419 (98.5)	659 (52.7)	

^a^Missing data in the population not in the sample and in the survey sample means that sums do not add to the population of general practitioners (N=3326), general practices (N=1674), and study sample (N=425).

^b^Population data from General Practitioners’ Organization [65].

^c^Population calculated from data by the Danish Health Data Authority [67].

^d^Municipality types based on the definition by Statistics Denmark [72].

### Measurements Based on the TAM

Table 2 presents the mean values (SD) of the 4 dimensions and the items from TAM. On a 5-point Likert scale, the highest mean value was PEOU 3.76 (SD 0.86) and ATT 3.48 (SD 0.92), thus indicating that respondents were confident that they, for instance, can use video consultations to manage type 2 diabetes and that the technology was a good idea. The mean values for PU 2.99 (SD 0.96) and BI to use 3.06 (SD 1.04) were similar, and the answers averaged around neither agreeing nor disagreeing. Across the studied dimensions and items, the data variability around the mean of the study sample was approximately 1 point on a 5-point Likert scale.

**Table 2:** Means and internal consistency of items in the research model (N=425).

Item		Participants, n (%)	Values, mean (SD)	Cronbach α
**PU^a^**				
	PU1: can *improve* my treatment	389 (91.5)	2.70 (0.97)	.86
	PU2: can make my treatment more *effective*	397 (93.4)	3.01 (1.07)	.78
	PU3: can make my treatment *easier*	396 (93.2)	3.24 (1.13)	.85
	PU: all usability items	379 (89.2)	2.99 (0.96)	.88
**PEOU^b^**				
	PEOU1: *learning* to use was (would be) easy	417 (98.1)	3.99 (0.95)	.85
	PEOU2: (would be) *easy to get* software to do what I need	401 (94.4)	3.81 (0.98)	.84
	PEOU3: (would be) easy to *master*	412 (96.9)	3.91 (0.91)	.83
	PEOU4: (would be) easy to *use*	372 (87.5)	3.28 (1.1)	.92
	PEOU: all ease of use items	359 (84.5)	3.76 (0.86)	.89
**ATT^c^**				
	ATT1: using is a *good idea*	409 (96.2)	3.29 (1.15)	.63
	ATT2: using is *unpleasant*	398 (93.6)	2.04 (0.96)	.92
	ATT3: using is *beneficial*	397 (93.4)	3.13 (1.09)	.68
	ATT: all attitude items^d^	380 (89.4)	3.48 (0.92)	.83
	ATT1+3: ATT excluding ATT2	393 (92.5)	3.21 (1.08)	.92
**BI^e^ to use**				
	BI1: intend to use as *often as possible*	403 (94.8)	2.66 (1.12)	.82
	BI2: even when possible, *do not intend* to use	404 (95.1)	2.61 (1.2)	.88
	BI3: would *use to the extent possible*	402 (94.6)	3.12 (1.12)	.78
	BI: all intention items^f^	383 (90.1)	3.06 (1.04)	.88

^a^PU: perceived usefulness.

^b^PEOU: perceived ease of use.

^c^ATT: attitude.

^d^The mean represents all ATT variables with ATT2 reversed because of its negative wording.

^e^BI: behavioral intention.

^f^The mean represents all BI variables with BI2 reversed because of its negative wording.

The internal consistency of the items that comprise the 4 dimensions in TAM had Cronbach α >.8 (Table 2). Cronbach α values of ≥.7 indicate acceptable internal consistency. Although the internal consistency of attitude was .83, this value should be interpreted with caution. The right-hand column of Table 2 shows the effect of removing 1 of the 3 items on Cronbach α; that is, for the attitude dimension, the Cronbach α drops to .63 and .68 when removing items 1 and 2 and increases to .92 when removing item 3. In addition to attributing this change in internal consistency to this analytical finding, free-text remarks by some respondents indicated that the negative wording of item 3 could be confusing and challenging to answer. On the basis of logical reasoning [73] and to reflect the attitude dimension more accurately, we excluded item 2 from the subsequent analysis.

To determine the correct structural equation modeling estimation method, we calculated the skewness and kurtosis of all the measures to examine normality. The results showed a mild degree of skewness (ranging from −0.971 to 0.232) with moderate kurtosis (ranging from 2.134 to 3.841). Normality was further evaluated using the Mardia multivariate kurtosis test, in which all dimensions failed except attitude, thereby indicating nonnormally distributed measures (PU 20.4, *χ*^2^_1_=90.9, *P*<.001; PEOU 43.3, *χ*^2^_1_=694.6, *P*<.001; attitude 8.22, *χ*^2^_1_=0.3, *P*=.57; BI 17.9, *χ*^2^_1_=26.0, *P*<.001). As nonnormality invalidates the assumption for the maximum likelihood method of structural equation modeling estimation, we used Satorra-Bentler adjustments to relax the assumption of normality. The measures in TAM were also assessed for convergent validity and discriminant validity (Table 3).

The measures were further validated using a confirmatory factor analysis that showed factor loadings >0.7, except for the item PEOU4—*easy to use* (0.63). PEOU4 was also an outlier in terms of missing data, with 12.7% (53/425) of missing responses, leading to the suspicion that the data were not missing at random. We excluded PEOU4 from the analysis and ran a new confirmatory factor analysis, which had factor loadings ranging from 0.77 to 0.92, thereby confirming that the latent variables of TAM were explained by the observed variables. Goodness-of-fit indices confirmed that the confirmatory factor analysis was a good fit for the data (*χ*^2^_38_=51.5, *χ*^2^/*df*=1.4; *P*=.07; root mean squared error of approximation 0.033 [recommended value <0.05]; standardized root mean square residual 0.024 [recommended value <0.08]; comparative fit index 0.995 [recommended value >0.95]) [74]. The final research model included data from 76.9% (327/425) of respondents.

**Table 3:** Correlations between dimensions and items in the research model.

Item		PU^a^	PEOU^b^	ATT^c^	BI^d^
**PU**					
	PU1	0.731	0.213	0.702	0.640
	PU2	0.824	0.335	0.761	0.700
	PU3	0.747	0.328	0.785	0.701
**PEOU**					
	PEOU1	0.204	0.803	0.250	0.378
	PEOU2	0.181	0.826	0.265	0.359
	PEOU3	0.224	0.853	0.301	0.410
	PEOU4	0.477	0.607	0.553	0.551
**ATT**					
	ATT1	0.800	0.419	0.844	0.789
	ATT3	0.801	0.369	0.844	0.765
**BI**					
	BI1	0.703	0.454	0.754	0.813
	BI2	0.613	0.441	0.668	0.711
	BI3	0.709	0.426	0.750	0.773

^a^PU: perceived usefulness.

^b^PEOU: perceived ease of use.

^c^ATT: attitude.

^d^BI: behavioral intention.

### Hypothesis Testing

We used structural equation modeling to analyze our hypotheses and the final research model. The goodness-of-fit indices model showed an acceptable fit (Table 4).

**Figure 2:**
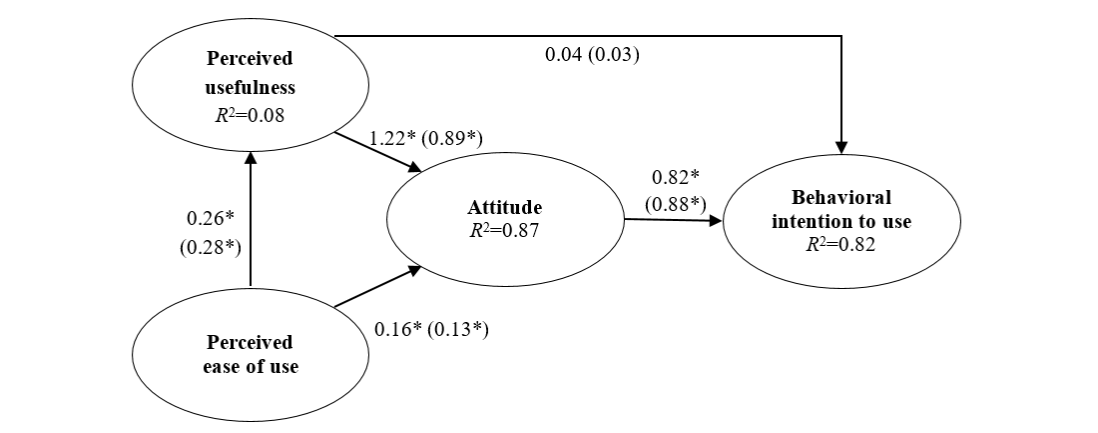
Results of structural equation modeling, unstandardized (and standardized) coefficients. *P<.001.

Analysis of the research model using unstandardized coefficients (Figure 2; Table 5) showed that the original paths of the model were significant (*P*<.005), except for the path from PU to BI to use (*P*=.84). PEOU had a positive influence on PU (β=.26, 95% CI 0.14-0.38) and attitude (β=.16, 95% CI 0.08-0.24). PU had a positive influence on attitude (β=1.22, 95% CI 1.09-1.36). The influence of attitude and PU on BI to use was also positive (β=.82, 95% CI 0.52-1.12; β=.04, −0.38 to 0.47); however, the latter was statistically insignificant. The calculated *R*^2^ values (Figure 2) showed that 82% of the variance in BI to use was explained by attitude and PEOU, with attitude having the strongest influence. Standardized coefficients showed similar results (Figure 2; Table 6) and indicated that the strongest relationship existed between PU and attitude and between attitude and BI.

**Table 4:** Fit indices for structural equation modeling estimation.

Fit index	Structural equation modeling model with Satorra-Bentler	Recommended value [74,75]
Chi-square (*df*)	63.59 (39)	N/A^a^
Chi-square/*df*	1.63	<3.0
*P* value>chi-square (*df*)	0.008	>0.05
Root mean squared error of approximation	0.044	<0.05
Comparative fit index	0.991	>0.95
Tucker-Lewis index	0.987	>0.95
Standardized root mean square residual	0.036	<0.08

^a^N/A: not applicable (the literature on structural equation modeling does not recommend a value).

**Table 5:** Structural equation modeling estimation, unstandardized coefficients^a^.

Path	β coefficient	*z* value	*P* value	95% CI
PEOU^b^→PU^c^	.26	4.26	<.001	0.14 to 0.38
PU→attitude	1.22	17.44	<.001	1.09 to 1.36
PEOU→attitude	.16	4.01	<.001	0.08 to 0.24
PU→BI^d^	.04	0.20	.84	−0.38 to 0.47
Attitude→BI	.82	5.35	<.001	0.52 to 1.12

^a^Satorra-Bentler adjusted; unstandardized coefficients.

^b^PEOU: perceived ease of use.

^c^PU: perceived usefulness.

^d^BI: behavioral intention.

**Table 6:** Structural equation modeling estimation, standardized coefficients^a^.

Path	β coefficient	*z* value	*P* value	95% CI
PEOU^b^→PU^c^	.28	4.09	<.001	0.15 to 0.42
PU→attitude	.89	38.19	<.001	0.84 to 0.94
PEOU→attitude	.13	4.09	<.001	0.07 to 0.19
PU→BI^d^	.03	0.19	.85	−0.31 to 0.37
Attitude→BI	.88	5.54	<.001	0.57 to 1.19

^a^Satorra-Bentler adjusted; standardized coefficients.

^b^PEOU: perceived ease of use.

^c^PU: perceived usefulness.

^d^BI: behavioral intention.

## Discussion

### Principal Findings and Comparison With Prior Work

To explore the potential of using video consultations to provide type 2 diabetes care in general practice, we used insights from technology adoption [36-40] to systematically elicit the technology acceptance of general practitioners. From our survey of Danish general practitioners, we found support for 4 of the 5 research hypotheses (standardized and unstandardized path coefficients).

First, our findings suggest that PU is the primary driver of a positive attitude toward using video consultations to provide type 2 diabetes in general practice (hypothesis 3 accepted: unstandardized β=1.22, 95% CI 1.09-1.36). Similarly, earlier research in general practice found that this relationship appeared to be highly important [53,54]. The unstandardized path coefficient indicates that increasing the PU of the technology by 1 unit will increase the attitude by 1.22 units, given the other variables in the model. The standardized coefficient (β=.89, 95% 0.84-0.94) shows that a change of 1 SD in PU leads to an increase by 0.89 SDs in attitude. Second, attitude toward the technology is positively influenced by general practitioners’ PEOU (hypothesis 2 accepted: unstandardized β=.16, 95% CI 0.08-0.24); however, the impact is lower than that for PU (β=1.22 vs β=.16). This finding mirrors previous studies that found that PU, not PEOU, is the primary driver of users’ attitudes toward health care technology. A reason is that ease of use is not necessarily a sufficiently large benefit to offset the difficulties of integrating new technology into established work routines [76]. Another reason is that the importance of a technology that is easy to use tends to decrease with general technology use [38,55,56].

Third, our analysis confirmed the expectation that general practitioners’ PU of video consultations would be positively influenced by their ratings of PEOU (hypothesis 1 accepted: unstandardized β=.26,95% CI 0.14-0.38). This mirrors findings from studies of other types of health care technology [50-52]. The relatively small impact of PEOU may be attributed to the high education level of Danish general practitioners who use IT technologies daily to deliver care, such as electronic patient records, and thus have a basic level of IT skills that could be speculated to give them confidence in learning new technologies.

Fourth, the BI to use video consultations to provide type 2 diabetes was positively influenced by the attitude toward the technology (hypothesis 5 accepted: unstandardized β=.82, 95% CI 0.52-1.12). This particular relationship has also been found in other studies in the domain of primary health care [23,60,61]. Attitude is a central driver that corresponds to other influential theories of behavior change, such as the theory of planned behavior [77]. Fifth, our research model links PU to BI to use; however, the positive influence was statistically insignificant (hypothesis 4 rejected: unstandardized β=.04, −0.38 to 0.47). Compared with the impact of attitude, the influence of the PU of video consultations was also less influential (β=.82 vs β=.04). Studies from general practice generally report that PU has a positive influence on BI to use [35,57-59]. However, these studies do not include the attitude dimension from the original model [39] in their research models and, thus, do not address the relative importance. Our findings indicate that the BI to use video consultations for type 2 diabetes care is primarily the result of the positive impact PU has on attitude.

By studying chronic care in our context—type 2 diabetes—our research findings contribute to an emerging literature on video consultations in general practice that has hitherto mostly studied acute or subacute or out-of-hours care [17,25,31]. A major strength of the study is that the findings build on TAM, which is a robust model [36,37] with good predictive power for health technology use [38]. The findings are also supported by goodness-of-fit tests, showing that the research model has an acceptable fit for structural equation modeling estimation. A strength of our analysis is that it did not rely on the assumption that the measures were normally distributed as we used the Satorra-Bentler adjustments in the structural equation modeling.

### Practical Implications

The potential of using video consultations in general practice to deliver chronic disease management is promising [1,10,11] and could fundamentally change how the primary care sector delivers care [12,19]. Type 2 diabetes is a chronic disease for which video consultations in general practice are particularly relevant [43-45] because, as a new care model, it can deliver high-quality, efficient care [46,47] at a time when the prevalence of diabetes is increasing [48]. Our findings (standardized and unstandardized path coefficients in the research model) indicate that the strongest positive relationships are between PU and attitude and between attitude and BI to use. This suggests that if a policy maker wants to increase general practitioners’ use of video consultations to provide type 2 diabetes care, they must ensure that the technology is useful in general practice as it will have a positive influence on their attitude, which, in turn, will positively affect their intention to use the technology. Policy makers interested in scaling up video consultations could benefit from looking into the items of the dimensions that constitute the research model. For example, to improve PU, policy makers should find solutions to three questions: how can it be ensured that video consultations (1) improve treatment, (2) make treatment more effective, and (3) make treatment easier?

Relatedly, our findings provide suggestions for mitigating change hesitance, which remains a barrier to implementing video consultations in routine practice [21]. As research shows that working in larger practices—but not individual characteristics such as age or sex—increases the likelihood that a general practitioner uses video consultation [22,23], it appears relevant to explore the perceptions of small and large practices separately. Using the example of PU, small and large practices may differ in the ways in which video consultations can improve and make treatment easier. These insights are important as data from, for example, the Danish Health Authority show a decrease in the use of video consultations in general practice from 2020 to 2021 [78], which suggests that general practitioners use the technology but also that it is not yet a regular work routine in general practice. Moreover, continuous improvement of the technology and its use in practice is central as there is a risk that this new care model increases general practitioner workload, and there may be a need to allocate more resources to implement digital-first pathways [14]. To the latter end, research finds that training facilitates the implementation of video consultations in routine practice [20].

### Limitations

Two modifications were made to the original TAM, underlining the final research model. First, an item (attitude item 2) was removed as it decreased the Cronbach α of the attitude dimension. Another item (PEOU4) was dropped because of the low factor loading from the confirmatory factor analysis. To assess the extent to which removing these items changed the findings, a structural equation modeling estimation, including these items, was performed, which showed path coefficients very similar to our final model, thereby supporting the accuracy of the final structural equation model. Second, structural equation modeling estimations were not performed with all respondents as those skipping questions were omitted. Running a structural equation modeling estimation that included respondents with missing answers resulted in similar path coefficients but had poorer goodness of fit. The final research model met the recommended indices of the goodness of fit but failed the chi-square test. Failing the chi-square test is a known issue with structural equation modeling, which, similar to our study, has a high number of respondents and survey answers that are not normally distributed [75]. The issue of nonnormality was addressed using Satorra-Bentler adjustments.

With the widespread research validation of TAM in combination with acceptable goodness-of-fit indices, the final research model is considered valid. However, as this study surveyed general practitioners from a tax-financed health care system, the findings may be most generalizable to countries with similar health care systems such as the English National Health System. Some authors also raise the concern that the original TAM and later extensions lack precision in health care because of their inability to consider the influence of external variables and barriers to technology acceptance [36] such as psychological ownership of IT [79] or social norms [55]. Nevertheless, for the purposes of this study, the research model was kept simple for 2 main reasons. First, findings from health care that extend TAM only result in a relatively modest increase in explanatory power [55]. Second, getting general practitioners to answer surveys is difficult [43], and including other variables to increase the precision a little would likely come at the expense of a lower response rate. More questions also increased the risk of respondent fatigue and missing answers.

The relatively low response rate of 12.8% of all 3326 Danish general practitioners increased the risk of selection bias. Nevertheless, it improved confidence in the findings that the individual characteristics of the sample of general practitioners were comparable with the population, and the share of respondents in the sample who used video consultations was similar to that of other sources [78]. This finding supports the generalizability of our results. The difficulty in getting Danish general practitioners to participate in survey research is an explanation as they operate as for-profit firms and are often on a tight schedule [62]. The survey was also distributed during the COVID-19 pandemic when other surveys of general practitioners had similar low response rates [22,43,80]. It could be speculated that general practitioners with the strongest positive or negative attitudes toward technology were more likely to participate. Univariate normality tests of the items in the attitude dimension, as mentioned previously, showed that the respondents’ attitudes were relatively normally distributed and did not only represent the most negative or positive attitudes toward video consultations used for diabetes care.

The study design was cross-sectional and, thus, only capable of capturing the views of general practitioners at the time of data collection. Although the cross-sectional design is standard in most studies on TAM [37,38], longitudinal studies are generally recommended to assess changes over time to make study findings more robust. Collecting data on the variables in TAM from the same source (ie, general practitioners) makes common method bias [81] a potential risk in the study. However, common method bias is of modest importance here as the research model asks about the intention to use rather than actual use.

### Conclusions

This study explored the potential of using video consultations to provide type 2 diabetes care in general practice by eliciting the technology acceptance of a representative survey sample of Danish general practitioners. On the basis of TAM, our study suggests 2 main drivers: PU positively affects attitude toward using video consultations for diabetes care, and attitude positively affects the BI to use the technology. For policy makers interested in scaling up general practitioners’ use of video consultations to provide diabetes care, our findings indicate that they should emphasize how the technology can improve treatment and make it more effective and easier. To this end, policy makers may need to explore what these aspects of usefulness mean to general practitioners working in different organizational contexts.

## Abbreviations

BI: behavioral intention
PEOU: perceived ease of use
PU: perceived usefulness
TAM: technology acceptance model
